# Alkali metal doping of black phosphorus monolayer for ultrasensitive capture and detection of nitrogen dioxide

**DOI:** 10.1038/s41598-020-80343-9

**Published:** 2021-01-12

**Authors:** Azam Marjani, Mehdi Ghambarian, Mohammad Ghashghaee

**Affiliations:** 1 Department for Management of Science and Technology Development, Ton Duc Thang University, Ho Chi Minh City, Viet Nam; 2 Faculty of Applied Sciences, Ton Duc Thang University, Ho Chi Minh City, Viet Nam; 3 Gas Conversion Department, Faculty of Petrochemicals, Iran Polymer and Petrochemical Institute, P.O. Box 14975-112, Tehran, Iran; 4 Department of Petrochemical Synthesis, Faculty of Petrochemicals, Iran Polymer and Petrochemical Institute, P.O. Box 14975-112, Tehran, Iran

**Keywords:** Environmental sciences, Natural hazards, Chemistry, Energy science and technology, Engineering, Materials science, Nanoscience and technology, Physics

## Abstract

Black phosphorus nanostructures have recently sparked substantial research interest for the rational development of novel chemosensors and nanodevices. For the first time, the influence of alkali metal doping of black phosphorus monolayer (BP) on its capabilities for nitrogen dioxide (NO_2_) capture and monitoring is discussed. Four different nanostructures including BP, Li-BP, Na-BP, and K-BP were evaluated; it was found that the adsorption configuration on Li-BP was different from others such that the NO_2_ molecule preferred a vertical stabilization rather than a parallel configuration with respect to the surface. The efficiency for the detection increased in the sequence of Na-BP < BP < K-BP < Li-BP, with the most significant improvement of + 95.2% in the case of Li doping. The Na-BP demonstrated the most compelling capacity (54 times higher than BP) for NO_2_ capture and catalysis (− 24.36 kcal/mol at HSE06/TZVP). Furthermore, the K-doped device was appropriate for both nitrogen dioxide adsorption and sensing while also providing the highest work function sensitivity (55.4%), which was much higher than that of BP (10.4%).

## Introduction

In recent decades, chemosensors and biosensors have been studied intensely due to the increasing importance of safety precautions and environmental protections. Sensors have been used in many industries for the detection of harmful compounds. Nitrogen dioxide (NO_2_) with a biting odor is considered as one of the most vital noxious gases in terms of air pollution. Also, it is used for the commercial production of nitric acid. Hence, modern society has a strong motivation to explore more sensitive NO_2_ detectors for controlling its impact on the environment and protection of human health and safety^1^. Various types of sensors are actively studied for this purpose. Some well-established classes of detectors including solid electrolytes, electrochemical sensors, graphene-based systems, as well as metal oxides have been introduced^2^.

Novel nanostructured and two-dimensional (2D) materials are actively pursued toward different optical and sensing applications^3–11^. Investigations of black phosphorus structures have been carried out by many researchers after its successful fabrication for different optical, biomedical, and environmental applications^12–17^. These 2D nanomaterials have outstanding heat as well as electron conductivities due to their great anisotropic properties^18–21^. Moreover, black phosphorus possess appropriate chemical and fire resistance^22,23^.

With regard to NO_2_, efficient detectors have been introduced, which have been reviewed elsewhere^1^. The research on such sensors is of great interest in different research groups exploring the properties of 2D materials^18,24–27^. Black phosphorus itself has provided physisorption capabilities for several gases, such as NO_2_^18,28^. However, BP has been recorded to outperform molybdenum disulfide (MoS_2_) for the nitrogen dioxide analysis in terms of the sensitivity threshold and quick regeneration^29^. An increased conductance sensitivity down to 5 ppb of nitrogen dioxide has been recorded for the multilayer BP detector^30^. In another study of nitrogen dioxide adsorption capabilities, Si embedding into graphene was quite effectual^31^. another study has shown that graphene/NiO heterostructures were helpful for NO_2_ sensing^32^. Similarly, the NiO-decorated BP slab has shown (116 times) stronger adsorption relative to the pristine layer for nitrogen dioxide capture and catalysis^18^. Aluminum decoration of BP has also shown useful for the capture of this toxic molecule with the adsorption energy of 3.96 eV^33^. Recently, the SnO monolayer slab has been presented to be auspicious for nitrogen dioxide capture and monitoring^34^. Among 2D nanomaterials, gallium nitride demonstrated significant bandgap alterations of 1.65 eV after the exposure to nitrogen dioxide^35^. Recently, the effectiveness of defect and ZnO species incorporation into the BP monolayer was shown for the NO_2_ monitoring and removal, respectively^19^.

Doping is a useful tool for the modulation of the anisotropic characteristics of the 2D materials for the sensing applications. For instance, Zhou et al. have found that the embedding of transition metals, particularly Ti and Au, can significantly enhance the chemical reactivity of graphene, thus leading to activation of the NO_2_ molecule for the graphene-based catalysis applications^36^. One may reasonably postulate that the effective doping of black phosphorus can enhance its capabilities toward the adsorption of NO_2_ molecule. In spite of the intriguing characteristics of black phosphorus, still little is known about the possible effects of such modifications on either the adsorption strength or sensitivity. In the same line, we explored the effects of alkali metal (Li, Na, and K) doping of BP monolayer on the sensitivity to the NO_2_ molecule. The nitrogen dioxide detection using black phosphorene has not been studied conclusively. The current research study deals with investigation of different BP structures in terms of the electronic as well as energetic properties for nitrogen dioxide adsorption.

As will be shown in the following, the suggested modifications can make substantial improvements in both the adsorption and sensing of NO_2_ on black phosphorene depending on the alkali metal employed. In a broader sense, the reported data would help supply a deeper understanding for the rational development of novel phosphorene-based nanomaterials for the adsorption and sensing of gaseous pollutants.

## Results and discussion

Several BP-based sensors have been inspected here, which include the pristine (BP) and alkali-doped M-BP nanomaterials (M = Li, Na, K). The methodology for the construction of the sensors is given in the Supplementary Information. Several modifiers, including transition metal (TM) elements, have been examined in the phosphorene functionalization and doping, as reviewed elsewhere^13^. Here, we have chosen the lightest members of the alkali metals, being among the ten most abundant elements in Earth crust^37^. As such, the modifications at hand would be less expensive and environmentally benign.

**Figure 1:**
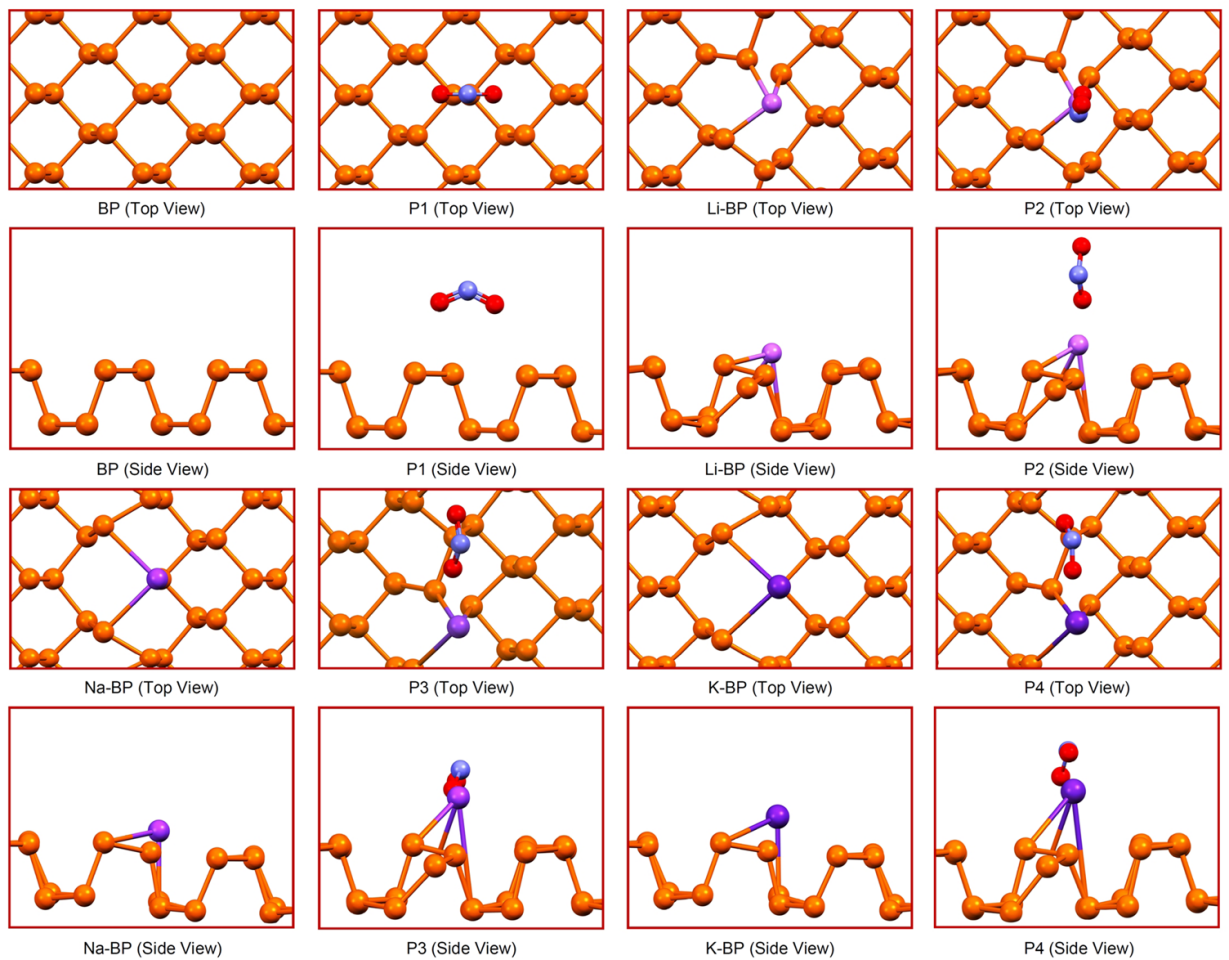
Relaxed configurations of the intact and working chemosensors obtained at the PBE/SVP computational level. The color indicators include orange for the P atoms, red for the O atoms, blue for the N atoms, and purple for the alkali metal (Li, Na, K) atoms. The images were drawn using Mercury 3.6.

The sensors with optimized geometries at the rest and operating conditions are shown in Fig. 1. From Fig. 1, we observe that the nitrogen dioxide molecule interacted through its two O atoms with the P atoms of the unmodified pristine layer while stabilizing in the armchair orientation with the O–N–O plane (P1). The stabilization of the NO_2_ molecule on the M-doped sensors was different. Notably, the related structure with Li-BP was the most different such that the molecule preferred a vertical arrangement with respect to the surface while interacting through an O atom (P2). This observation is in correspondence with the absence of the 3d orbitals in lithium and the lower protrusion of the Li atom compared to the other dopants (vide infra). The NO_2_ molecule on both Na-BP and K-BP was optimized more similarly to P1 except that the O–N–O angle was stabilized in the zigzag direction, with one of the oxygen atoms tending to approach the dopant center (P3 and P4). In both cases, the adsorption of NO_2_ molecule also led to the more projection of the metal atom away from the surface (vide infra); but, the solid matrix integrity was not lost in either case owing to the small distortions.

The formation energy values for the M-BP materials were calculated as follows:^21,38^

1 $$E_{{{\text{form}}}} = \, (E_{{{\text{M}} - {\text{BP}}}} + \mu_{{\text{P}}} ) \, {-} \, (E_{{{\text{BP}}}} + \mu_{{\text{M}}} ).$$

Here, *E*_BP_ and *E*_M-BP_ denote the total energies of the unmodified and alkali-doped BP slabs.; the symbol *µ*_P_ denotes the chemical potential of phosphorus. The pure phosphorene chemical potential was considered in this study. The symbol *µ*_M_ refers to the chemical potential of the M dopant. The energetics of the nitrogen dioxide molecule on various phosphorene-based structures was determined as follows:^21,39–41^

2 $$\Delta E_{{{\text{ads}}}} = E_{{{\text{op}}}} {-} \, \left( {E_{{{\text{gas}}}} + E_{{{\text{sensor}}}} } \right)$$

in which the subscript op refers to the adsorption configuration (the operating device), and gas denotes the nitrogen dioxide molecule.

The energetic data have been supplied in Table 1. The formation energy for the incorporation of alkali metal was predicted to be negative in all cases indicating that the incorporation of these metal elements into the structure would be highly favorable, and high doping concentrations are plausible thermodynamically. The sequence of Na-BP < K-BP < Li-BP was found for the energetic favorability of the modification. In spite of the fact that the adsorption of nitrogen dioxide on the pristine layer was weak, the gaseous molecule was almost tightly chemisorbed on the alkali-modified sensor, and the adsorption strength was sequenced as BP < Li-BP < K-BP < Na-BP (up to − 24.36 kcal/mol at HSE06/TZVP). The equilibrium distance for the NO_2_ molecule varied in the range of 0.31–2.79 Å, inversely correlating with the adsorption strength (Table 1). It was concluded that the NO_2_ adsorption strength had been increased 54 times upon sodium doping. Such enhancement implies that the alkali-doping is a useful means of increasing the capability of black phosphorene for NO_2_ capture.

**Table 1:** Adsorption distances (*d*, Å), formation energies (*E*_form_, eV), and the corresponding sorption energies (∆*E*_ads_, kcal/mol) of the NO_2_ molecule on the original and modified sensors at the HSE06/TZVP level of theory.

Sensor	*d*	*E*_form_	∆*E*_ads_
BP	2.79	–	− 0.45
Li-BP	1.82	− 1.63	− 9.68
Na-BP	0.31	− 1.17	− 24.36
K-BP	0.50	− 1.27	− 20.59

One can see from the geometrical data that the average distance for the M–P bonds increased in the order of Li-BP < Na-BP < K-BP (Table 2). Furthermore, the adsorption of NO_2_ led to an elongation of the *r*(M–P) values by 7.1%, 16.2%, and 13.4%, correlating with the adsorption strength. In the same line, the *r*(N–O) values increased slightly from 1.20 Å for the free molecule to 1.21, 1.23, 1.25, and 1.24 Å in P1, P2, P3, and P4, respectively. The geometrical features demonstrate that the stronger the adsorption, the shorter the adsorption distance, and the longer the M–P and N–O bonds at the adsorption site.

**Table 2:** Various specifications of the investigated sensors at the HSE06/TZVP level: the mean M–P bond length [*r*(M–P), Å], the mean N–O bond length [*r*(N–O), Å], the efficiency of the analysis (*ε*, %), the regeneration time of the sensor (*τ*, fs–s), the chemical potential factor (*μ*, eV), the global hardness of the structure (*η*, eV), and the electrophilicity indicator (*ω*, eV).

Structure	*r*(M–P)	*r*(N–O)	*ε*	*τ*	*μ*	*η*	*ω*
BP	–	–	–	–	5.00	0.73	17.18
P1	–	1.21	40.5	19.3 fs	5.07	0.60	21.57
Li-BP	2.81 ± 0.45	–	–	–	4.53	0.83	12.37
P2	3.01 ± 0.57	1.23 ± 0.03	79.1	14.8 ns	5.47	0.53	28.07
Na-BP	3.08 ± 0.17	–	–	–	4.43	0.71	13.88
P3	3.58 ± 0.70	1.25	22.4	33.2 s	5.28	0.78	17.89
K-BP	3.37 ± 0.06	–	–	–	3.97	0.64	12.32
P4	3.82 ± 0.62	1.24 ± 0.01	43.5	132.1 ms	5.29	0.64	21.71

The efficiency was defined using Eq. (7). Also, the visible light radiation at 343 K was considered for the calculation of the recovery time.

**Figure 2:**
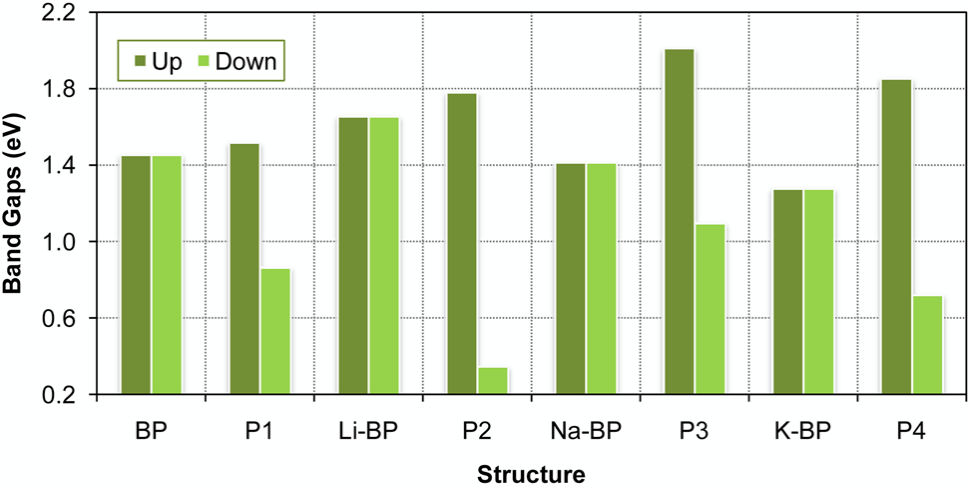
Electronic bandgaps of the unmodified and alkali-modified sensors at the off state and working conditions for nitrogen dioxide detection at the HSE06/TZVP computational level.

The induced magnetic moment in the operating mode was found to be 1.0 *µ*_B_ due to the stabilization of the nitrogen dioxide molecule on the surface. The spin moment (*µ*_s_) contribution from nitrogen dioxide was sequenced as P3 (0.03 *µ*_B_) < P4 (0.27 *µ*_B_) < P2 (0.63 *µ*_B_) < P1 (0.93 *µ*_B_). The charge gaps of the non-magnetic structures were determined as follows:

3 $$E_{{\text{g}}} = E_{{{\text{LUCO}}}} {-}E_{{{\text{HOCO}}}} ,$$

where *E*_LUCO_ refers to the lowest unoccupied crystal orbital (LUCO) energy level with the unit of eV. Also, *E*_HOCO_ denotes the highest occupied crystal orbital (HOCO) energy level (eV). Similarly, the spin-conserving gaps for both up and down spin channels could be determined using the following equations:^15^

4 $$E_{{{\text{g}} \uparrow }} = E_{{{\text{LUCO}} \uparrow }} {-}E_{{{\text{SOCO}} \uparrow }}$$

and

5 $$E_{{{\text{g}} \downarrow }} = E_{{{\text{LUCO}} \downarrow }} {-}E_{{{\text{SOCO}} \downarrow }}$$

in which *E*_SOCO_ denotes the single occupied crystal orbital (SOCO) energy level (eV). The nanosensor signal is determined by the alteration in the electrical conductance defined as follows:^15,42,43^

6 $${ }\sigma = {{AT}}^{{3/2}} {\text{exp}}\left( {\frac{{{{{-}E}}_{{\text{g}}} }}{{{\text{2}{k}}_{{\text{B}}} {{T}}}}} \right)$$

in which *T* signifies the working temperature (K), *k*_B_ signifies the Boltzmann constant (eV K^–1^), and *A* represents the constant of proportionality (in electrons m^–3^ K^–3/2^). The sensors bandgaps at the off state as well as during operation are provided in Fig. 2. As evinced by these charts, all sensors kept their semiconducting nature through modification and operation, and no half-metallic behavior was induced. The bandgap of the pristine layer was obtained as 1.46 eV, which indicates that there is a great agreement between the obtained value and the experimental range (1.0–1.5 eV) for the single-layer BP nanodevice^44^. This observation confirms the suitability of the method applied here^15,38^.

**Figure 3:**
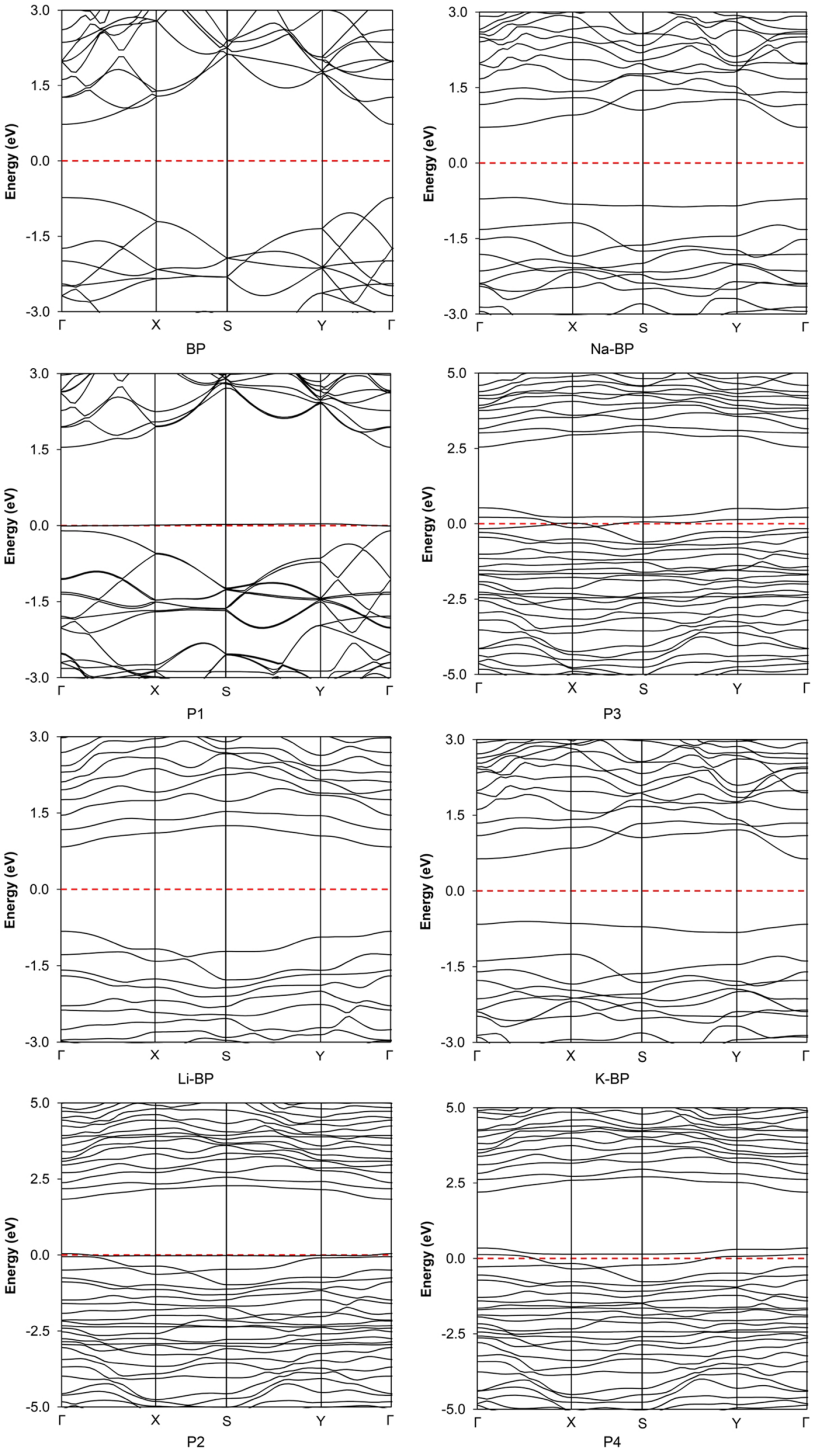
Band structures of the unmodified and alkali-modified sensors at the off and operating states at the HSE06/TZVP computational level. The outputs have been obtained from Burai 1.3.

The original bandgap (BP) was slightly expanded after lithium doping, but it was slightly shrunk after sodium and potassium doping (Fig. 2). However, it should be pointed out that the amount of bandgap in the up-spin channel was enlarged with the NO_2_ sensitivity, while it was decreased in the down-spin gap after the interaction of the analyte for all sensors. A huge variation in the bandgap was observed in the case of Li doping compared to the other cases, which implied the higher sensitivity of this sensor to the NO_2_ molecule. The electronic band structures of the sensors are presented in Fig. 3. The determined band structure for the pristine phosphorene was fully consistent with the Brillouin zone (BZ) data reported in the literature^45^, indicating the existence of a direct bandgap at the Γ point of the BZ^15^. A similar type of (direct) bandgap was obtained in the case of Li-BP and Na-BP. Nonetheless, the electronic band structure for the K-BP nanodevice exhibited an indirect X → Y nature. In terms of band alignment, the energy levels of the conduction band minimum (CBM) and the valence band maximum (VBM) both shifted upward upon alkali doping with the magnitude of the changes following the sequence of Li-BP < Na-BP < K-BP. The relative energy position of the Fermi level is discussed under the work function (vide infra). When the type of semiconduction was concerned, almost all metal impurities were neutral, thus retaining the intrinsic behavior of the original BP material intact. However, the analyte behaved as an acceptor and led to a p-type semiconductor in all operating cases.

The detector quality or efficiency was evaluated from the relative alterations of the bandgap:^18^

7 $$\varepsilon = { 100}\left( {\frac{{\left| {E_{g} {-}E_{0} } \right|}}{{E_{g} }}} \right)$$

in which *ε* represents the detector efficiency, while the initial state of the sensor was indicated by 0 and the symbol *E*_g_ is the bandgap at the operating conditions^18^. Based on this definition, the amount of *E*_g_ is negative when there is a shortening bandgap due to the interactions with the nitrogen dioxide. Table 2 reports the central operating data for the unmodified and alkali-doped sensors for the NO_2_ molecule analysis^19^. The absolute efficiency changed in the order of Na-BP < BP < K-BP < Li-BP, indicating the efficiency changes relative to the unmodified BP of + 95.2, − 44.6, and + 7.4% with the Li, Na, and K doping, respectively. Therefore, the Li-doped sensor has registered the highest efficiency (79.1%) for NO_2_ detection. So, it could be concluded that the pristine phosphorene became more capable of NO_2_ monitoring and removal with the incorporation of K and Li in its structure. On the basis of the intermediate adsorption energy (− 9.68 kcal/mol) released on Li-BP, it would be an ideal choice for NO_2_ detection (vide infra).

The sensors reusability is one of the most critical parameters in order to appraise its performance. Conventional transition state theory (TST) was applied to the calculation of the recovery time using the amount of adsorption energy:^43,46^

8 $$\tau = \nu_{0}^{{{-}1}} {\text{exp}}\left( {\frac{{{-}\Delta {{E}}_{{{\text{ads}}}} }}{{{{k}}_{{\text{B}}} {{T}}}}} \right)$$

in which Δ*E*_ads_ shows the adsorption energy (kcal/mol) determined from Eq. (2), and *ν*_0_ refers to the attempt frequency (s^−1^)^18^. The calculated data provided in Table 2 delineate the speed of regeneration. Although BP provided the lowest recovery time (19.3 fs), it should be mentioned that the obtained recovery period was extremely short for stable monitoring. Furthermore, the alkali-doped sensors were capable of being regenerated under visible radiation at 343 K. Interestingly, the most sensitive sensor (Li-BP) had a recovery time of 14.8 ns at these conditions. Even the calculated recovery time in the slowest case (33.2 s for Na-BP) was quite reasonable compared to the performance of novel tellurene and borophene materials^47,48^. Therefore, the findings point to the conclusion that the alkali doping can adjust the electronic properties of the phosphorene layer to be a sensitive and reusable sensor for nitrogen dioxide detection and monitoring. In addition, the Na-doped material would be more appropriate for NO_2_ removal and catalysis owing to the relatively high retention time and the stronger adsorption. Moreover, the K-BP material would be considered as an ultrasensitive sensor with reasonable reusability at mild conditions.

Table 2 shows more indicators for the evaluation of the sensors in terms of reactivity of the structures. The indicators were selected based on Koopman’s theorem^43,49,50^. The chemical potential is the first indicator, and can be expressed as follows:^15^

9 $$\mu = \, {-}\chi ,$$

10 $$\chi = \, \left( {I + A} \right)/{2} \approx {-}\left( {E_{{{\text{HOCO}}}} + E_{{{\text{LUCO}}}} } \right)/{2}$$

in which *I* represents the ionization potential, *χ* is the electronegativity, and *A* signifies the electron affinity. Another expression can be given for the magnetic complexes:^18,51,52^

11 $$\chi = \, {-}[(E_{{{\text{SOCO}} \uparrow }} + E_{{{\text{LUCO}} \uparrow }} ) \, + \, (E_{{{\text{SOCO}} \downarrow }} + E_{{{\text{LUCO}} \downarrow }} )]/{4}{\text{.}}$$

Also, the following equations were used for the estimation of the global hardness (Eq. 12), the spin potential at zero net spin transfer (Eq. 13), and the electrophilicity (Eq. 14):^18^

12 $$\eta = \, \left( {E_{{{\text{LUCO}}}} {-}E_{{{\text{HOCO}}}} } \right)/{2,}$$

13 $$\eta = \, [(E_{{{\text{LUCO}} \uparrow }} + E_{{{\text{LUCO}} \downarrow }} ) \, {-} \, (E_{{{\text{SOCO}} \uparrow }} + E_{{{\text{SOCO}} \downarrow }} )]/{4,}$$

14 $$\omega = \mu^{{2}} /\left( {{2}\eta } \right).$$

Table 2 contains such indicators for the four sensors at the HSE06/TZVP computational level^15^. The data showed that the chemical potential and electrophilicity values increased with the adsorption of the NO_2_ molecule. As indicated, the sensor efficiency correlated well with the electrophilicity of the operating sensor.

**Figure 4:**
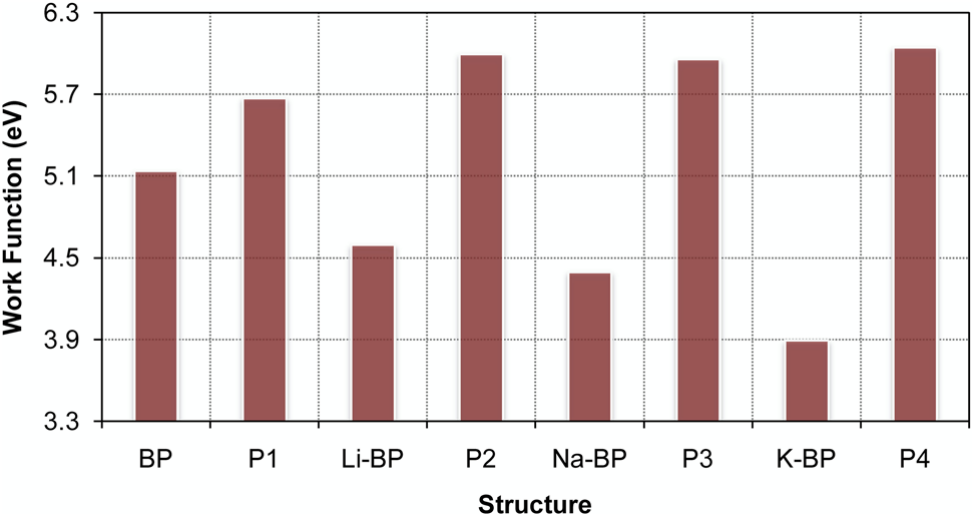
Work function sensitivity chart of the phosphorene-based materials for nitrogen dioxide capture and monitoring at the HSE06/TZVP computational level.

Another consideration is that the adsorption of nitrogen dioxide may alter the work function of the chemiresistive nanodevice. In such cases, the material would be a work function detector. Equation (15) was used for the evaluation of the changes in the work function during the detection process:^19^

15 $$\varphi = E_{{{\text{vac}}}} - E_{{\text{F}}} ,$$

where *E*_F_ denotes the Fermi energy level, and *E*_vac_ is the vacuum energy (the electrostatic potential in the vacuum)^15^. The obtained magnitudes are shown in Fig. 4. The base-case work function was 5.14 eV at the HSE06/TZVP level of theory, which was in excellent agreement with the theoretical magnitude of 5.03 eV^53^ and the experimental measurement of 5.30 eV^54^ for a single-layer phosphorene. The work function of the sensor itself was decreased in the order of K-BP < Na-BP < Li-BP < BP. Moreover, the adsorption of NO_2_ resulted in an enhancement in the work function of all sensors. These changes were sequenced as BP (10.4%) < Li-BP (30.5%) < Na-BP (35.6%) < K-BP (55.4%), indicating that the K-BP sensor would be an excellent work function sensor (5.3 times better than the pristine BP at the HSE06/TZVP) for the NO_2_ detection. Meanwhile, we note that the Na-BP has shown quite high work function sensitivity to NO_2_ while requiring higher temperatures for a fast recovery. Higher sensitivity and obvious reusability for nitrogen dioxide detection were obtained with the Li-doped phosphorene sensor.

**Figure 5:**
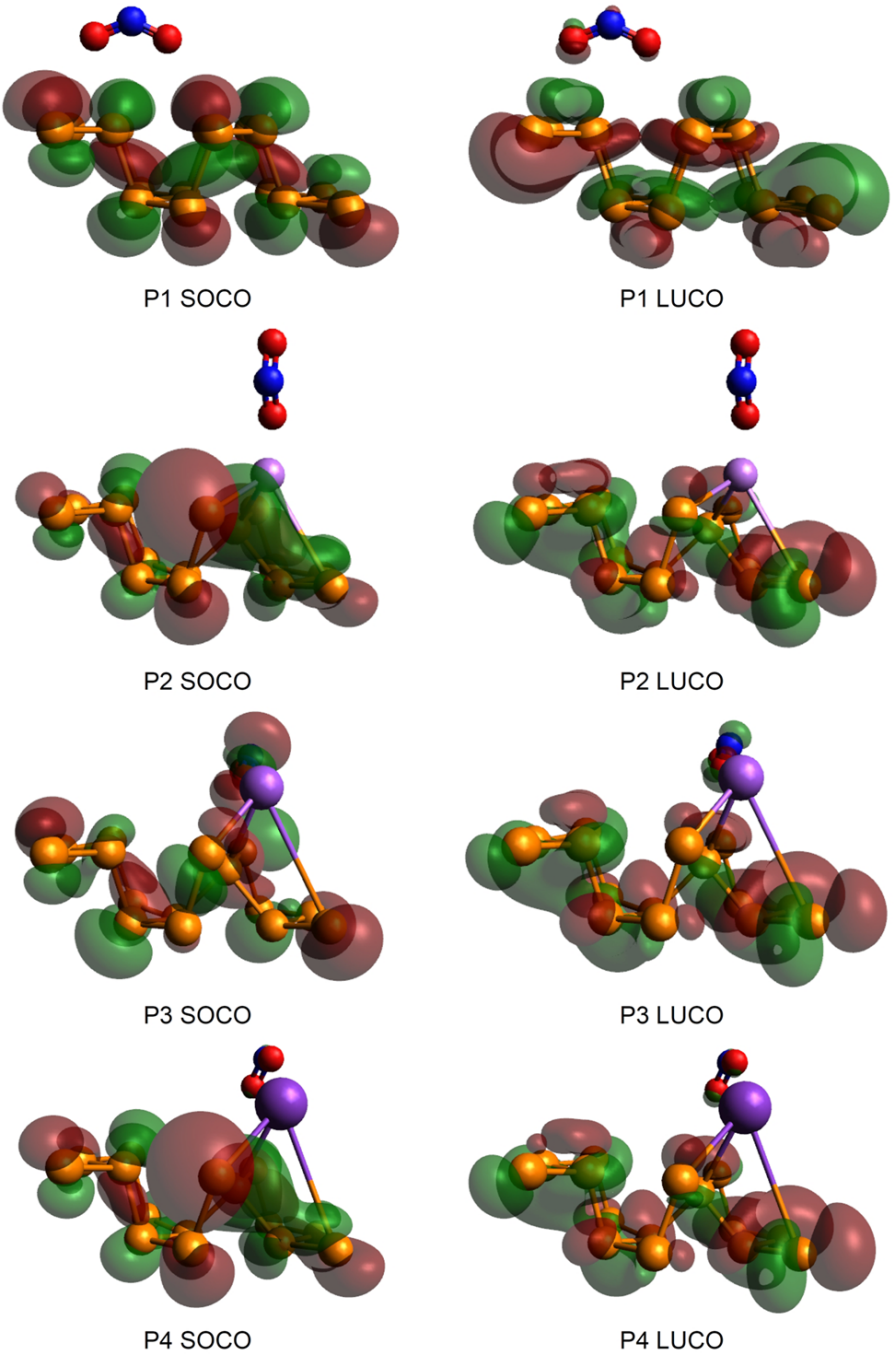
HOCO/LUCO dispersions of the working chemosensors at the HSE06/TZVP computational level. The orbitals have been drawn in Avogadro 1.2.0.

The frontier molecular orbital (FMO) dispersions of the adsorption adducts are illustrated in Fig. 5. The alkali doping changed the orbital distribution, while the density localization did not occur precisely at the dopant center. Specifically, the HOCO densities of the Li-BP and K-BP sensors were changed substantially. The nitrogen dioxide molecule was involved mostly in the LUCO of P1; however, a negligible contribution in HOCO could be found for the analyte. Over the alkali-modified sensors, the NO_2_ molecule showed the lowest contribution to the FMO with the Li-BP surface (P2). In terms of Na-BP performance (P3), the amount of HOCO electron density located on the analyte was more significant, which also exhibited an apparent LUCO involvement by this fragment. Finally, the nitrogen dioxide adsorption on K-BP directed to a weak delocalization of LUCO toward this fragment with almost no density localization in the case of HOCO. These observations qualitatively confirm the sequence of adsorption energies explained above.

**Figure 6:**
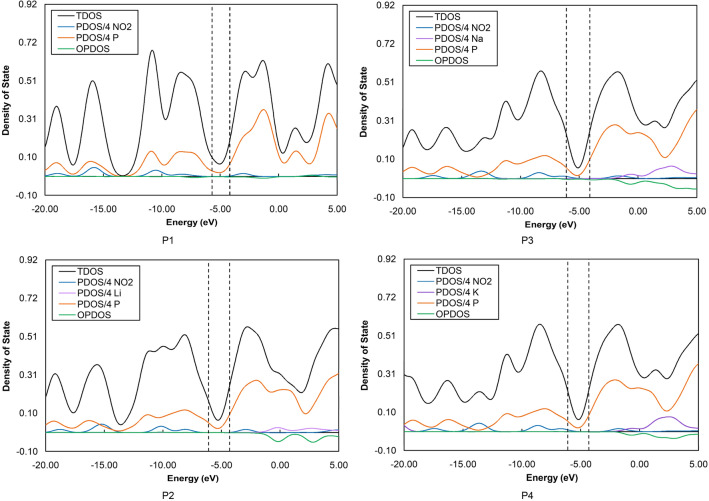
Projected (PDOS) and total (TDOS) profiles of the density of states for nitrogen dioxide monitoring with the four BP-based chemiresistive nanomaterials at the HSE06/TZVP computational level. The spectra have been obtained from Multiwfn 3.3.8.

The alkali-doped phosphorene structures can be discussed in terms of the projected (PDOS) and total (TDOS) density of states, as shown in Fig. 6 for the four sensors after the exposure to nitrogen dioxide. Also shown in Fig. 6 are the overlap population (OPDOS) curves of the NO_2_ molecule at the surface. As one can see in these plots, the partial density plots of the NO_2_ molecule were almost the same in the P1 and P2 configurations, due mainly to the relatively small involvement of the (NO_2_) analyte in the FMO distributions (vide supra). In the P3 and P4 structures, however, the corresponding PDOS pattern upshifted by ca. 1.3 eV, thus leading to a more pronounced contribution to the lower edge of the bandgap. This observation further supports our discussion of the FMO distributions. Further in the same line, the OPDOS curve in P2 indicated the non-bonding nature over an almost wide energy range around the Fermi level. Moving to P3, however, we observed anti-bonding behavior near the Fermi level. The orbitals of nitrogen dioxide molecule and phosphorus atoms were slightly hybridized at − 8.0 eV in the bonding regions of P2. Similar behavior was observed at − 8.2 and − 19.0 eV in the case of P1. Such observation was not the case for the rest of the sensors. Overall, these explanations describe how the most sensitive sensors behave differently for NO_2_ detection.

## Conclusion

In summary, this article studied for the first time the comparative influence of alkali metal doping of the pristine BP layer on its performance in terms of elimination and detection of nitrogen dioxide with the aid of periodic quantum-chemical calculations. While nitrogen dioxide was stabilized in a horizontal configuration aligned in the black phosphorene armchair direction and the zigzag directions of Na-BP and K-BP, the lowest-energy configuration on Li-BP was different such that the molecule preferred a vertical arrangement with an oxygen atom pointing to the surface. The formation energies indicated the feasibility of alkali doping at high concentrations following the order of Na-BP < K-BP < Li-BP. However, the Na-BP displayed the strongest analyte chemisorption (− 24.36 kcal/mol at HSE06/TZVP). Significant correlations were found between the adsorption distance and the M–P and N–O bond length alterations with the adsorption strength. For all nanosensors investigated here, the bandgap changed oppositely depending on the spin channel. While the type of bandgap was retained after Li and Na doping, an indirect X → Y transfer preference was observed in the K-BP band structure. The Li-doped phosphorene material showed the highest sensitivity (79.1%) toward the NO_2_ molecule (increased by 95.2% compared to the pristine BP). Interestingly, the most sensitive material in this series (Li-BP) had an optimal recovery time of 14.8 ns at 343 K. Meanwhile, the Na-BP material was found to be more appropriate for nitrogen dioxide capture and catalysis. On the grounds of the global indicators, a connection could be established between the responsivity and the electrophilicity at the working conditions. While BP was the least effective for NO_2_ removal, the K-doped material was applicable for nitrogen dioxide removal and detection, while also turning out to provide the highest work function sensitivity (55.4%) for the NO_2_ detection. In summary, we may conclude that the Li-doped monolayer can be considered as an ultrasensitive and recoverable NO_2_ chemosensor. Furthermore, the potassium embedding in the BP pristine layer can transform it into a dual-purpose device for both nitrogen dioxide removal/catalysis and sensing.

## Methods

Periodic DFT computations were conducted^19^ in the environments of CP2K^55^, NWChem 6.5^56^, Multiwfn 3.3.8^57^, and Burai 1.3 software^58^. The pictorial outputs were obtained with Mercury 3.6^59^ and Avogadro 1.2.0.^60^. Further details of the computational approach are given in the Supplementary Information.

## Supplementary Information

Supplementary Information.
