# Supplementary material for: Alkali metal doping of black phosphorus monolayer for ultrasensitive capture and detection of nitrogen dioxide
Source: Sci Rep. 2021 Jan 12;11:842. doi: 10.1038/s41598-020-80343-9 (PMC7804848; doi:10.1038/s41598-020-80343-9)
Supplement: Supplementary file 1 — Supplementary Information. [file 41598_2020_80343_MOESM1_ESM.pdf]

Supplementary Material

Alkali Metal Doping of Black Phosphorus Monolayer for Ultrasensitive Capture and Detection of Nitrogen Dioxide

Azam Marjani[1,2], Mehdi Ghambarian[3], Mohammad Ghashghaee[4,*]

[1] *Department for Management of Science and Technology Development, Ton Duc Thang University, Ho Chi Minh City, Viet Nam.* [2] *Faculty of Applied Sciences, Ton Duc Thang University, Ho Chi Minh City, Viet Nam.*

[3] *Gas Conversion Department, Faculty of Petrochemicals, Iran Polymer and Petrochemical Institute, P.O. Box 14975-112, Tehran, Iran*

[4] *Department of Petrochemical Synthesis, Faculty of Petrochemicals, Iran Polymer and Petrochemical Institute, P.O. Box 14975-112, Tehran, Iran*

* Corresponding author. Tel.: +98 21 48662481; fax: +98 21 44787032. E-mail address: m.ghashghaee@ippi.ac.ir.

Methods

The structure optimizations were implemented using the Perdew-Burke-Eernzerh (PBE) functional[1] combined with the SVP basis set.[2] The energetic data were calculated using the hybrid functional Heyd-Scuseria-Ernzerhof (HSE06)[3] coupled with TZVP.[4] The reference structure for bulk BP material was obtained from the established literature.[5] The modified sensors were obtained with the removal and subsequent replacement of one phosphorus atom with the alkali metal (Li, Na, and K) in a 2×2×1 phosphorene supercell. The supercell was allowed to relax toward the most stable energy structure. Finally, the NO_2 molecule was placed on the surface of each sensor, and the whole structure was optimized to the most stable configuration. A vacuum space of >20 Å was exerted to prevent possible mirror interactions between the adjacent layers in the perpendicular direction. Moreover, 20 *k*-points were taken into account for the band structure computations. Bandgap was calculated based on the Kohn–Sham (KS) frontier molecular orbital (FMO) theory at the same level of theory (HSE06/TZVP) taking the spin polarization into account where required.

Supplementary discussion

The M-doped analogs were obtained by the substitutional doping of the three alkali metal elements into the pristine layer in place of a single P atom. After the construction of the four nanosensors, the adsorption configurations were simulated by the optimization of the NO_2 molecule on their surface. The obtained structures were named P1, P2, P3, and P4, respectively.

References

1 Perdew, J. P., Burke, K. & Ernzerhof, M. Generalized Gradient Approximation Made Simple. *Phys. Rev. Lett.* **77**, 3865-3868, doi:10.1103/PhysRevLett.77.3865 (1996).

2 Schäfer, A., Horn, H. & Ahlrichs, R. Fully optimized contracted Gaussian basis sets for atoms Li to Kr. *J. Chem. Phys.* **97**, 2571-2577, doi:10.1063/1.463096 (1992).

3 Izmaylov, A. F., Scuseria, G. E. & Frisch, M. J. Efficient evaluation of short-range Hartree-Fock exchange in large molecules and periodic systems. *J. Chem. Phys.* **125**, 104103, doi:10.1063/1.2347713 (2006).

4 Schäfer, A., Huber, C. & Ahlrichs, R. Fully optimized contracted Gaussian basis sets of triple zeta valence quality for atoms Li to Kr. *J. Chem. Phys.* **100**, 5829-5835, doi:10.1063/1.467146 (1994).

5 Lange, S., Schmidt, P. & Nilges, T. Au_3SnP_7@Black Phosphorus: An Easy Access to Black Phosphorus. *Inorg. Chem.* **46**, 4028-4035, doi:10.1021/ic062192q (2007).
